# A Novel Noise Reduction Method of UAV Magnetic Survey Data Based on CEEMDAN, Permutation Entropy, Correlation Coefficient and Wavelet Threshold Denoising

**DOI:** 10.3390/e23101309

**Published:** 2021-10-06

**Authors:** Yaoxin Zheng, Shiyan Li, Kang Xing, Xiaojuan Zhang

**Affiliations:** 1 Aerospace Information Research Institute, Chinese Academy of Sciences, Beijing 100094, China; zhengyaoxin17@mails.ucas.ac.cn (Y.Z.); lishiyan18@mails.ucas.ac.cn (S.L.); xingkang19@mails.ucas.ac.cn (K.X.); 2 Key Laboratory of Electromagnetic Radiation and Sensing Technology, Chinese Academy of Sciences, Beijing 100190, China; 3 School of Electronic, Electrical and Communication Engineering, University of Chinese Academy of Sciences, Beijing 100049, China

**Keywords:** UAV magnetic survey, data processing, CEEMDAN, permutation entropy, correlation coefficient, wavelet threshold denoising

## Abstract

Despite the increased attention that has been given to the unmanned aerial vehicle (UAV)-based magnetic survey systems in the past decade, the processing of UAV magnetic data is still a tough task. In this paper, we propose a novel noise reduction method of UAV magnetic data based on complete ensemble empirical mode decomposition with adaptive noise (CEEMDAN), permutation entropy (PE), correlation coefficient and wavelet threshold denoising. The original signal is first decomposed into several intrinsic mode functions (IMFs) by CEEMDAN, and the PE of each IMF is calculated. Second, IMFs are divided into four categories according to the quartiles of PE, namely, noise IMFs, noise-dominant IMFs, signal-dominant IMFs, and signal IMFs. Then the noise IMFs are removed, and correlation coefficients are used to identify the real signal-dominant IMFs. Finally, the wavelet threshold denoising is applied to the real signal-dominant IMFs, the denoised signal can be obtained by combining the signal IMFs and the denoised IMFs. Both synthetic and field experiments are conducted to verify the effectiveness of the proposed method. The results show that the proposed method can eliminate the interference to a great extent, which lays a foundation for the further interpretation of UAV magnetic data.

## 1. Introduction

The past decade has seen a variety of applications conducted by unmanned aerial vehicles (UAVs) in many fields, e.g., archaeology, remote sensing, geological prospecting, and unexploded ordnance (UXO) detection [1]. Among these applications, the use of UAVs for magnetic surveys is a booming branch of research [2]. UAV magnetic surveys can cover a wider range with a higher efficiency compared with the traditional terrestrial magnetic surveys, and are also easy to operate, have a low-cost, and have a good safety profile compared with manned aircraft magnetic surveys [3]. In addition, UAV-based magnetic surveys can also be carried out in areas that are difficult to access or that would pose a potential hazard to operators (e.g., near active volcanoes), which means that some gaps in traditional magnetic surveys can now be studied [4,5].

A substantial body of research has accumulated on the integration of UAV magnetic survey systems; however, the processing of UAV magnetic data remains an open problem. Several attempts have been made to process UAV magnetic data, e.g., Malehmir et al. [6] used a median filter to process the spiky sample points, while data with severe noise were excluded. However, the quality of the collected data has not been evaluated. Walter et al. [7] investigated the periodic variations caused by the swing of magnetometers, spectral analysis and lowpass filter were applied to identify and remove the periodic signal, yet the target signal may be removed if its frequency overlaps with the periodic signal. Mu et al. [8] proposed a lowpass filter to remove the interference field; however, the cutoff frequency and the order of the filter need to be determined according to a priori knowledge. Similar methods were also used to process the multi-rotor UAV magnetic data, as described in [9,10]. Liu et al. [11] proposed an adaptive cancellation of geomagnetic background noise for magnetic anomaly detection. The system is regarded as a two-channel linear time-invariant (LTI) system, where the first sensor records the background noise as a reference, and the second sensor records the target signal and background noise at the same time. It should be noted that if the system is a single channel (i.e., a magnetometer), it will be difficult to use this method. Wang et al. [12] used higher-order statistics to suppress the interference of Gaussian colored noise in magnetotelluric data. However, if the noise is complex and no longer obeys Gaussian distribution, this method will not be able to suppress interference effectively. In addition, this method requires redundant data, which limits its further implementation. Overall, processing methods alone are not enough, since the collected data are usually non-stationary and easily affected by noise from many sources, e.g., interference generated by UAV platform, geological noise, industrial frequency interference, and instrument noise.

Empirical mode decomposition (EMD), proposed by Huang [13], is an adaptive time-frequency analysis method which is suitable for non-linear and non-stationary signals. EMD analyzes the signal according to the characteristics of the signal itself and does not need a basis function [14]. However, the boundary effects and mode mixing heavily impact the effect of EMD. To overwhelm these problems, a noise-assistant analysis method, i.e., ensemble empirical mode decomposition (EEMD), is proposed [15]. EEMD basically overcomes the mode mixing; however, two new problems, the difference in intrinsic mode function (IMF) numbers and the introduction of extra noise, have arisen. A complete ensemble empirical mode decomposition with adaptive white noise (CEEMDAN) is proposed to surmount these obstacles [16]. The CEEMDAN method can significantly reduce the reconstruction error and requires fewer iterations compared with EMD and EEMD. To date, CEEMDAN has been widely used in the field of non-linear and non-stationary signal processing, e.g., biological signal processing [17,18], wind speed forecasting [19,20], financial time series forecasting [21], gear fault diagnosis [22,23,24], underwater acoustic signal denoising [25,26], and structural damage localization and quantification [27].

To further study the characteristics of non-linear and non-stationary signals, permutation entropy (PE) and correlation coefficient (CC) are proposed to evaluate the complexity of obtained IMFs, and identify whether the IMFs require denoising, as noted in several previous studies [22,25,28,29]. In addition, wavelet threshold denoising is adopted as part of the combined method [17,25,26]. To the best of our knowledge, there have been no previous studies on UAV magnetic data denoising based on CEEMDAN. Moreover, the determination of noisy IMFs is generally based on the artificial threshold, which is not only difficult to achieve in practice, but also does not make full use of the characteristics of the signal itself.

In this paper, a novel noise reduction method for UAV magnetic data is proposed by taking advantage of CEEMDAN, PE, CC, and wavelet threshold denoising. The main contributions of the proposed method are as follows:

1. The adaptive decomposition algorithm, i.e., CEEMDAN, is applied to multi-rotor UAV magnetic data for the first time. The original data are decomposed into a set of IMF components with different scales.
2. The IMFs are divided into four categories, i.e., noise IMFs, noise-dominant IMFs, signal-dominant IMFs, and signal IMFs according to the quartiles of PE, which is completely determined by the characteristics of the signal itself without setting a threshold artificially.
3. The real signal-dominant IMFs are identified using CC, while the wavelet soft threshold denoising (WSTD) is applied to further suppress noise. Simulation results show that the signal-to-noise ratio (SNR) of the signal can be improved by about 16–20 dB after denoising by means of the proposed method.

This paper is organized as follows: Section 2 presents the relevant principles of CEEMDAN, PE, CC, and wavelet threshold denoising; the proposed noise reduction algorithm for UAV magnetic data is presented in Section 3; in Section 4 and Section 5, the proposed method is applied to both synthetic and real UAV magnetic data, respectively; Section 6 contains the conclusion of this paper.

## 2. Relevant Principles

### 2.1. Principles of EMD, EEMD, and CEEMDAN Algorithm

In this section, the mathematical principles of EMD, EEMD, and CEEMDAN are introduced, and the specific implementation steps, diagrams, and pseudocodes are given to better understand how these methods work.

#### 2.1.1. EMD Algorithm

EMD can adaptively decompose the original signal $s\left(t\right)$ into several IMFs and a residue. The IMF meets the following two conditions: (a) the number of extremum points and zero-crossing points must be equal or not exceed one, and (b) the mean value of the upper envelope formed by the local maximum points and the lower envelope formed by the local minimum points is zero. The procedure of EMD is summarized as follows [30]:

Step 1: Identify all the extremum points of the original signal $s\left(t\right)$ and define the upper and lower envelope $u\left(t\right)$ and $l\left(t\right)$, respectively, using a cubic spline interpolation.
Step 2: Calculate the mean envelope of the upper and lower envelope.
  (1) $$m\left(t\right)=\frac{1}{2}\left(u\left(t\right)+l\left(t\right)\right).$$
Step 3: Subtract the mean envelope from *s*(*t*) to obtain the first intermediate signal.
  (2) $$I_{1}\left(t\right)=s\left(t\right)-m\left(t\right).$$
Step 4: If $I_{1}\left(t\right)$ satisfies the criteria of the IMF, then define $IMF_{1}=I_{1}\left(t\right)$, otherwise treat $I_{1}\left(t\right)$ as the new signal and repeat the above procedure k times until $I_{k+1}\left(t\right)$ satisfies the IMF conditions. The acquisition of IMF usually requires several iterations. To finish the iteration, the stopping criterion is defined as follows:
  (3) $$S_{D}=\sum_{i=1}^{n}\frac{{\left|I_{k}\left(i\right)-I_{k+1}\left(i\right)\right|}^{2}}{I_{k}^{2}\left(i\right)},$$
  where n is the length of the intermediate signal. The iteration will be stopped when $S_{D}<\delta$ (in this study δ is set to 0.3).
Step 5: Let $r_{1}\left(t\right)=s\left(t\right)-IMF_{1}$, treat $r_{1}\left(t\right)$ as the new signal and repeat Step 1–4 to obtain the next IMF, until $r_{N}\left(t\right)$ becomes either a constant or a monotonic function. Finally, the original signal *s*(*t*) after EMD can be expressed as:
  (4) $$s\left(t\right)=\sum_{i=1}^{N}IMF_{i}+r_{N}\left(t\right),$$
  where *N* is the number of IMFs, and $r_{N}\left(t\right)$ is the final residue.

**Figure 1:**
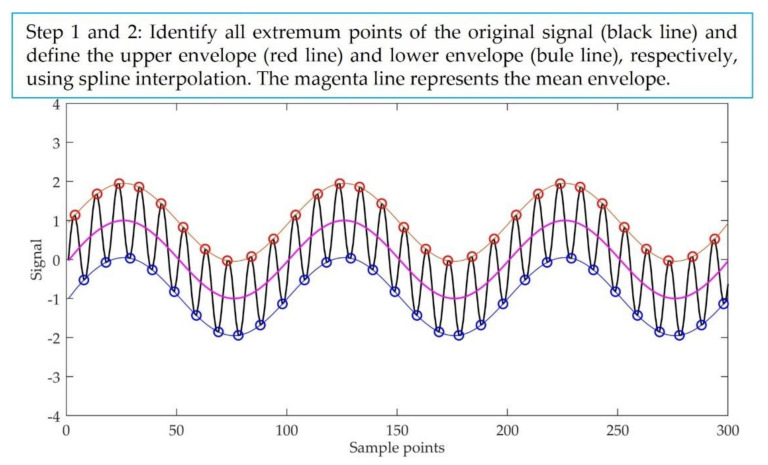

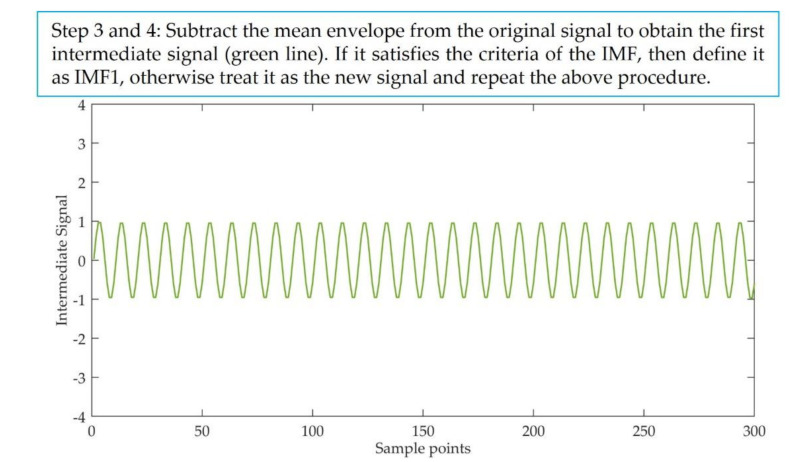
The schematic diagram of the main steps of EMD.

The pseudocode of EMD is described in detail in Algorithm 1. Figure 1 is the schematic diagram of the main steps of EMD.
**Algorithm 1**: EMD**Input:**   The original signal x.**Output:**  Several IMF and a residue, i.e., $IMF_{i},\left(i=1,2,\ldots ,n\right)$ and r.1: **function** EMD (x, ResidueThreshold, $S_{DT}$)2:  IMF←03:  *i*←04:  
*N*
$\leftarrow length\left(x\right)$
5:  *residue*
←∞
6:  **while** *residue*> ResidueThreshold
**do**7:   *i*←i+18:   $x_{i}\leftarrow x-\sum_{i}IMF_{i}$
9:   
$S_{D}\leftarrow \infty$
10:   **while**
$S_{D}>S_{DT}$
**do**11:    **for**
*j* = 1→*j* = N **do**12:     [$LocalMax_{ij}$, $IndMax_{ij}$] $\leftarrow \max\left(x_{i}\right)$
13:     [$LocalMin_{ij}$, $IndMin_{ij}$] $\leftarrow \min\left(x_{i}\right)$
14:    **end for**15:   $UpperEnv_{i}\leftarrow spline\left(IndMax_{i},LocalMax_{i},1:length\left(x_{i}\right)\right)$
16:   $LowerEnv_{i}\leftarrow spline\left(IndMin_{i},LocalMin_{i},1:length\left(x_{i}\right)\right)$
17:   $LocalMeanEnv_{i}\leftarrow \left(UpperEnv_{i}+LowerEnv_{i}\right)/2$
18:   $x_{i}\leftarrow x_{i}-LocalMeanEnv_{i}$
19:   $S_{D}\leftarrow \sum [\frac{{\left(xTemp-x_{i}\right)}^{2}}{xTemp^{2}}]$
20:   **end while**21:  
$IMF_{i}\leftarrow x_{i}$
22:  *residue*
$\leftarrow mean\left(x-\sum_{i}IMF_{i}\right)$
23:  **end while**24:  **return IMF and residue**25: **end function**

The effect of EMD is easily affected by mode mixing, i.e., signals of different feature scales appear in the same IMF, or signals with the same feature scale are dispersed into different IMFs [31]. This problem not only decreases the decomposition efficiency but also degrades the subsequent denoising performance.

#### 2.1.2. EEMD Algorithm

The EEMD algorithm is proposed to eliminate the mode mixing of EMD. The procedure of EEMD is summarized as follows [17,26]:

Step 1: Different Gaussian white noise signals with zero mean and unit variance $n_{i}\left(t\right)$ are added to the original signal $s\left(t\right)$ to obtain a set of new signals
  (5) $$s_{i}\left(t\right)=s\left(t\right)+n_{i}\left(t\right),\;i=1,2,\ldots ,P.$$
Step 2: Decompose each $s_{i}\left(t\right)$ by EMD to obtain $IMF_{ik}$, where k=1,2,…,N denotes the number of IMFs.
Step 3: Average the $IMF_{ik}$ to obtain the EEMD mode
  (6) $$IMF_{k}=\frac{1}{P}\sum_{i=1}^{P}IMF_{ik}.$$

The pseudocode of EEMD is described in detail in Algorithm 2.
**Algorithm 2**: EEMD**Input:**  The original signal x, the amplitude of the added Gaussian noise σ, and the number of ensemble trials m.**Output**:  Several IMF and a residue, i.e., $IMF_{k},\left(k=1,2,\ldots ,p\right)$ and r.1:  **function** EEMD (x, σ, m)2:   IMF←03:   *r*
←0
4:   **for** *i* = 1 →i = m
**do**5:    $n_{i}\leftarrow N\left(\sigma *\mathrm{std}\left(x\right),1\right)$
6:    
$x_{i}\leftarrow x+n_{i}$
7:    EMD ($x_{i}$)8:    
$r\leftarrow mean\left(r_{m}\right)$
9:   **end for**10:   **for**
*k* = 1 →k = p **do**11:     $IMF_{k}\leftarrow$ $mean(IMF_{i,k}$)12:   **end for**13:   
residue←r
14:   **return IMF and residue**15:  **end function**

**Figure 2:**
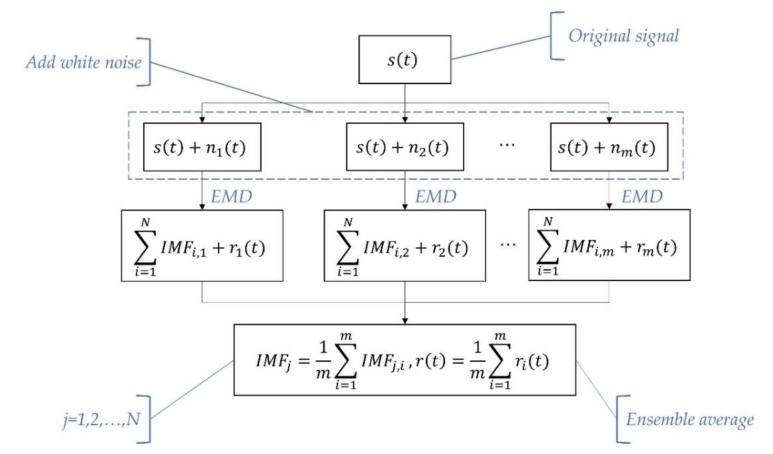
The flow chart of EEMD.

The flow chart of EEMD is shown in Figure 2.

#### 2.1.3. CEEMDAN Algorithm

Since the number of ensemble average is finite, a reconstruction error still exists in the result of EEMD. CEEMDAN can effectively overcome the mode mixing, with the reconstruction error and computational cost significantly reduced. The procedure of CEEMDAN is summarized as follows [25,26,32]:

Step 1: The white noise $\varepsilon_{0}n_{i}\left(t\right)$ is added to the original signal $s\left(t\right)$, and the first IMF of CEEMDAN is obtained by calculating the ensemble average:
  (7) $$IMF_{1}=\frac{1}{N}\sum_{i=1}^{N}E_{1}\left(s\left(t\right)+\varepsilon_{0}n_{i}\left(t\right)\right),$$
  where $E_{n}\left(*\right)$ is defined as the *n*th mode component of EMD.
Step 2: The first residual component can be obtained
  (8) $$r_{1}\left(t\right)=s\left(t\right)-IMF_{1}.$$
Step 3: Construct the new signal
  (9) $$s_{1}\left(t\right)=r_{1}\left(t\right)+E_{1}\left(n_{i}\left(t\right)\right),$$
  and decompose it by EMD. The second mode component can be obtained:
  (10) $$IMF_{2}=\frac{1}{N}\sum_{i=1}^{N}E_{1}\left(r_{1}\left(t\right)+E_{1}\left(n_{i}\left(t\right)\right)\right).$$
Step 4: The *n*th residual signal and the (*n*+1)th IMF can be obtained according to the process of Step 3
  (11) $$r_{n}\left(t\right)=r_{n-1}\left(t\right)-IMF_{n}\left(t\right),$$
  (12) $$IMF_{n+1}=\frac{1}{N}\sum_{i=1}^{N}E_{1}\left(r_{n}\left(t\right)+E_{n}\left(n_{i}\left(t\right)\right)\right).$$
Step 5: Repeat Step 4 until the residual signal is no longer decomposed. The original signal can be expressed as
  (13) $$s\left(t\right)=\sum_{n=1}^{K}IMF_{n}+r\left(t\right),$$
  where *K* is the number of IMFs by CEEMDAN, and $r\left(t\right)$ is the final residual mode.

The pseudocode of CEEMDAN is described in detail in Algorithm 3.
**Algorithm 3**: CEEMDAN**Input:** The original signal x, the amplitude of the added Gaussian noise σ, and the number of ensemble trials m.**Output:** Several IMF and a residue, i.e., $IMF_{k},\left(k=1,2,\ldots ,p\right)$ and r.1:  **function** CEEMDAN (x, σ, m)2:   IMF←03:   
*residue*
←0
4:   **for** *i* = 1 →i= m
**do**5:    
$n_{i}\leftarrow N\left(\sigma *\mathrm{std}\left(x\right),1\right)$
6:    
$x_{i}\leftarrow x+n_{i}$
7:    $IMF_{i}\leftarrow E_{1}$($x_{i}$)8:    
$IMF_{1}\leftarrow \frac{1}{m}\sum_{i}IMF_{i}$
9:   **end for**10:   
$r_{1}\leftarrow x-IMF_{1}$
11:   **for**
*k* = 2 →k = p **do**12:    
$IMF_{k}\leftarrow mean\left(E_{1}\left(r_{k-1}+E_{1}\left(n_{i}\right)\right)\right)$
13:    
$r_{k}\leftarrow r_{k-1}-IMF_{k}$
14:   **end for**15:   
$r\leftarrow r_{p}$
16:   **return IMF and residue**17:  **end function**18:19:  **function** $E_{1}$(x)20:   IMF←**EMD** (x)21:   
$IMF_{1}\leftarrow IMF\left(1,:\right)$
22:   **return**
$IMF_{1}$
23:  **end function**

**Figure 3:**
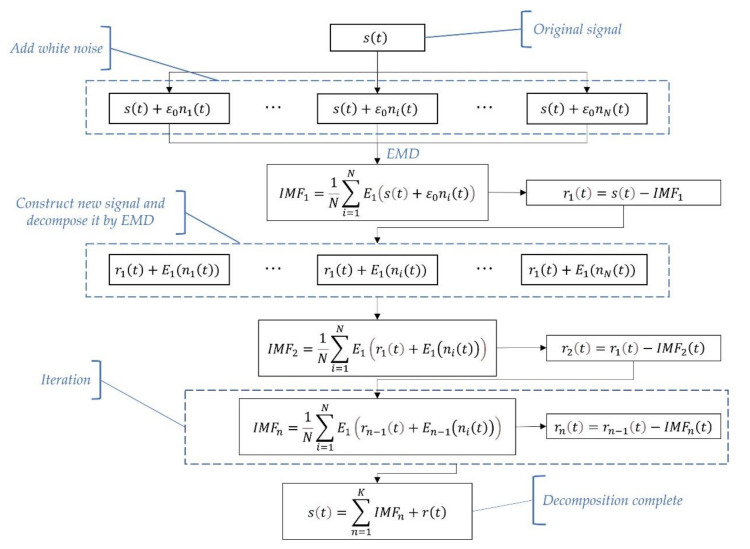
The flow chart of CEEMDAN algorithm.

The flow chart of CEEMDAN is shown in Figure 3.

### 2.2. Permutation Entropy

PE was initially introduced by Bandt and Pompe [33] as a tool for measuring the complexity of time series, the advantages of PE are its simplicity, fast calculation, better robustness, and strong anti-noise ability, which make it suitable for the feature extraction of non-linear data. The specific steps of PE are summarized as follows [23,29]:

Step 1: The first step in the calculation of permutation entropy requires extracting ordinal information from the time series. Given a time series $X=\left\{x_{1},x_{2},\ldots ,x_{N}\right\}$, *K* reconstructed time series can be obtained as:
  (14) $$\left\{\begin{array}{c} \begin{array}{c} \left\{x_{1},x_{1+\tau },\ldots ,x_{1+\left(m-1\right)\tau }\right\}\\ \vdots \end{array}\\ \left\{x_{j},x_{j+\tau },\ldots ,x_{j+\left(m-1\right)\tau }\right\}\\ \begin{array}{c} \vdots \\ \left\{x_{K},x_{K+\tau },\ldots ,x_{K+\left(m-1\right)\tau }\right\} \end{array} \end{array}\right.,$$
  where m and τ represent the embedding dimension and time delay, respectively. $K=N-\left(m-1\right)\tau$.
Step 2: For the *j*th reconstructed component, rearrange it in ascending order:
  (15) $$x_{j+r_{0}}\leq x_{j+r_{1}}\leq \ldots \leq x_{j+r_{m-2}}\leq x_{j+r_{m-1}},$$
Step 3: The permutation of (15) is defined as:
  (16) $$\pi_{j}=\left(r_{0},r_{1},\ldots ,r_{m-2},r_{m-1}\right).$$
Step 4: If the probabilities of each permutation are $\;P_{\pi_{1}},P_{\pi_{2}},\ldots ,P_{\pi_{K}}$, respectively. The PE of time series X is defined as
  (17) $$H_{pe}\left(m\right)=-\sum_{i=1}^{m!}P_{\pi_{i}}log_{2}P_{\pi_{i}}.$$
Step 5: PE can be normalized as
  (18) $$0\leq H_{PE}\left(m\right)=\frac{H_{pe}\left(m\right)}{log_{2}m!}\leq 1.$$

A simple example may help to clarify this concept. Assume a time series X= (1,5,3,4,2), the embedding dimension m is set to 3 and the time delay τ is set to 1. Three reconstructed time series can be obtained as: (1,5,3), (5,3,4), and (3,4,2). According to (16), the permutation for (1,5,3), (5,3,4), and (3,4,2) is (0,2,1), (1,2,0), and (2,0,1), respectively. The normalized permutation entropy can be obtained as: $PE=-\frac{1}{log_{2}3!}\left(\frac{1}{3}log_{2}\frac{1}{3}+\frac{1}{3}log_{2}\frac{1}{3}+\frac{1}{3}log_{2}\frac{1}{3}\right)=0.6131$. PE indicates the degree of randomness of the time series, i.e., the smaller the PE is, the simpler and more regular the time series is. The embedding dimension m and the time delay τ are two key parameters that affect the value of PE. In this paper, we set m=3 and τ=1 according to a previous study [29]. The pseudocode of PE is described in Algorithm 4.
**Algorithm 4:** PE [34]**Input:** The time series x, the embedding dimension m.**Output:** PE (x, m).**Define:** 
${\left(\pi^{m}\right)}_{k}$ is the *k*-th permutation of $\pi^{m}$, time delay τ = 1.1: **function** PE (x, m)2:  PE←03:  *N*←*length* (x)4:  
$c\leftarrow {\left\{0\right\}}_{m!}$
5:  
$p\leftarrow {\left\{0\right\}}_{m!}$
6:  
$\pi^{m}\leftarrow \left\{0,1,\ldots ,m-1\right\}$
7:  
$\Pi^{m}\leftarrow \left\{\pi^{m},{\left(\pi^{m}\right)}_{1},\ldots ,{\left(\pi^{m}\right)}_{m!-1}\right\}$
8:  **for** *j = 0*→j= *N*−m
**do**9:   
$x_{j}^{m}\leftarrow \left\{x_{j},x_{j+1},\ldots ,x_{j+m-1}\right\}$
10:   
$sort\left(x_{j}^{m},\pi^{m}\right)\to \left(y_{j}^{m},\pi_{j}^{m}\right)$
11:   **for**
*i = 0*→i = m!−1
**do**12:   **if** $\pi_{j}^{m}==\Pi_{i}^{m}$
**then**13:    
$c_{i}=c_{i}+1$
14:   **break**15:   **end for**16:  **end for**17: **for** k* = 0*→k = m!−1 do18:    
$p_{k}\leftarrow \frac{c_{k}}{\left(N-m-1\right)}$
19:   **if** $p_{k}>0$ then20:    
$PE=PE+\left(-p_{k}log_{2}p_{k}\right)$
21:   **end if**22:  **end for**23:  **return PE**24: **end function**

### 2.3. Correlation Coefficient

The correlation coefficient is a dimensionless index that is widely applied in multivariate statistics to represent the relationship between two groups of variables [28]. Its value ranges from −1 to 1. The larger the absolute value of the correlation coefficient is, the stronger the correlation between the two variables is. For the two groups of variables x and y, the correlation coefficient $\rho_{xy}$ is defined as follows [35]

(19) $$\rho_{xy}=\frac{cov\left(x,y\right)}{\sqrt{cov\left(x,x\right)\cdot cov\left(y,y\right)}},$$

where $cov\left(x,y\right)$ is the covariance of x and y, $cov\left(x,x\right)$ and $cov\left(y,y\right)$ are the variance of x and y, respectively. Therefore, the correlation coefficient can be expressed as

(20) $$\rho_{xy}=\frac{\sum_{i=1}^{n}\left(x_{i}-\bar{x}\right)\left(y_{i}-\bar{y}\right)}{\sqrt{\sum_{i=1}^{n}{\left(x_{i}-\bar{x}\right)}^{2}\cdot \sum_{i=1}^{n}{\left(y_{i}-\bar{y}\right)}^{2}}},$$

(21) $$\bar{x}=\frac{1}{n}\overset{n}{\underset{i=1}{\sum }}x_{i},\bar{y}=\frac{1}{n}\overset{n}{\underset{i=1}{\sum }}y_{i}.$$

### 2.4. Wavelet Threshold Denoising

The wavelet transform is an effective time-frequency analysis tool which has the characteristic of multi-resolution and has been widely used for signal processing [36,37]. For a one-dimensional noisy signal,

(22) $$s\left(t\right)=f\left(t\right)+e\left(t\right),\;t=0,1,\ldots ,n,\;$$

where $s\left(t\right)$, $f\left(t\right)$, and $e\left(t\right)$ are defined as the noisy signal, real signal, and Gaussian noise signal, respectively. The specific steps of wavelet-based denoising are as follows [25]:

Step 1: Proper wavelet basis function and decomposition level are selected to conduct wavelet decomposition on the noisy signal $s\left(t\right)$.
Step 2: The thresholds are estimated according to appropriate threshold selection criteria for the high-frequency coefficients at different decomposition scales.
Step 3: The low-frequency coefficients of decomposition and the threshold high-frequency coefficients are used to reconstruct signals.

The key to wavelet threshold denoising is the selection of the wavelet basis function and the threshold function. The db4 wavelet basis function and a soft threshold method are selected in this paper.

## 3. The Proposed Method for UAV Magnetic Data Denoising

**Figure 4:**
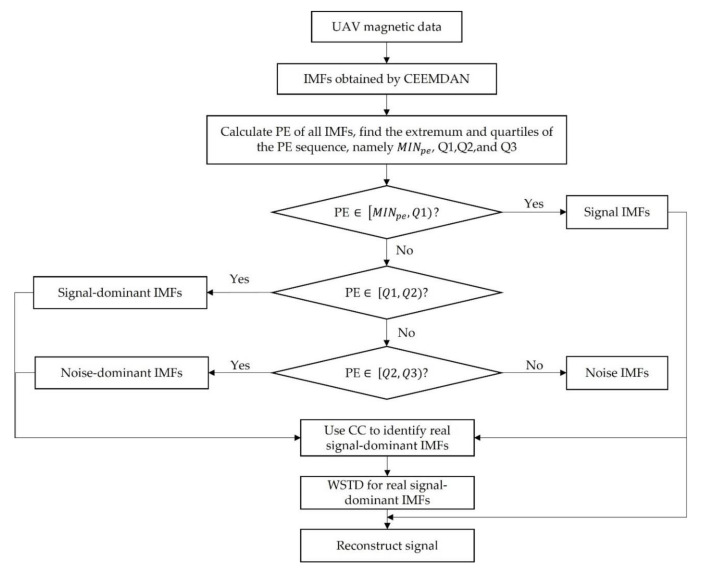
The flow chart of the proposed denoising algorithm for UAV magnetic data.

A denoising algorithm for UAV magnetic survey data based on CEEMDAN, PE, CC, and WSTD is proposed in this paper. The proposed method is based on the premise that there is a significant difference in complexity between the target signal and noise, so PE can be used to measure whether the IMF is dominated by target signal or noise. To avoid the influence of unreasonable threshold setting on subsequent processing, IMFs are divided into four categories by the quartiles of PE. The real signal-dominant IMFs are further confirmed by CC, and the noise is further suppressed by WSTD. The flow chart of the proposed method is shown in Figure 4. The specific procedures are summarized as follows:

Step 1: The original signal $s\left(t\right)$ is decomposed into several IMFs by CEEMDAN and arranged from high frequency to low frequency.
Step 2: Calculate the PE of all IMFs, the PE sequence is arranged in ascending order, and the extremum and the quartiles of the PE sequence are found, namely $MIN_{pe}$, *Q*1, *Q*2, *Q*3, and $MAX_{pe}$.
Step 3: Execute the judgement procedure: (1) if PE falls in the interval of $[MIN_{pe},\left.Q1\right)$, the corresponding IMFs are considered as signal IMFs and are preserved; (2) if PE falls in the interval of $[\left.Q1,Q2\right)$, the corresponding IMFs are defined as signal-dominant IMFs; (3) if PE falls in the interval of $[\left.Q2,Q3\right)$, the corresponding IMFs are defined as noise-dominant IMFs; (4) if PE falls in the interval of $[Q3,MAX_{pe}]$, the corresponding IMFs are defined as noise and are removed.
Step 4: The CCs between signal (noise)-dominant IMFs and the real signal which is constituted by signal IMFs are obtained. The median of the absolute value sequence of CCs is recorded as $M_{cc}$, and the IMFs corresponding to the CC greater than $M_{cc}$ are defined as the real signal-dominant IMFs.
Step 5: WSTD is applied to the real signal-dominant IMFs. The wavelet basis function and the decomposition level are db4 and 4, respectively.
Step 6: The denoised signal can be obtained by combining the signal IMFs and the denoised IMFs.

## 4. Synthetic Signal Denoising Experiment

### 4.1. Acquisition of Synthetic Signal

**Figure 5:**
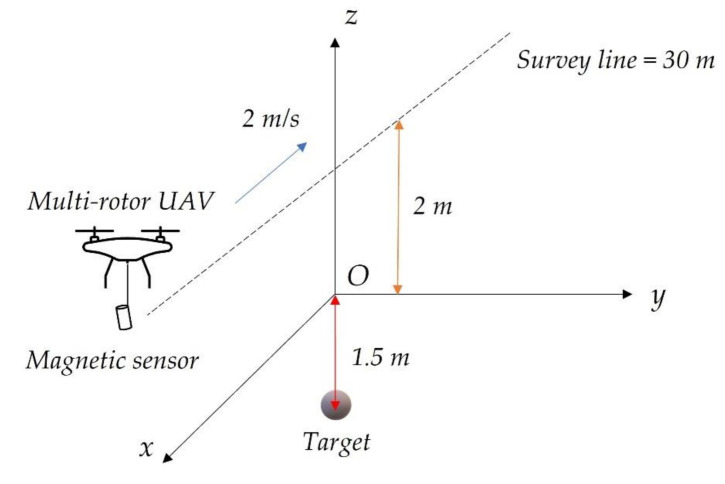
The magnetic target detection model.

Generally, the target signal of UAV magnetic surveys can be considered as a magnetic dipole, since the distance between sensors and the target is usually 2.5 times greater than the maximum dimension of the target [38]. The magnetic anomaly field generated by the target can be calculated by:

(23) $$B=\frac{\mu_{0}}{4\pi } [\frac{3\left(m\cdot r\right)r}{r^{5}}-\frac{m}{r^{3}}],$$

where $\mu_{0}$ is the permeability in vacuum, m is the dipole moment of the target, r is the displacement vector from the target to the measurement point, and $r=\left|r\right|$. As illustrated in Figure 5, the target center is located 1.5 m below the coordinate origin, with a magnetic moment $m=\left(-1.35,\;0.12,-0.78\right)A\cdot m^{2}$. The length of the survey line is 30 m, with a sampling interval of 0.02 m. The distance between the magnetic sensor and the ground is 2 m, and the speed of the UAV is 2 m/s. The geomagnetic field intensity is 56,000 nT, while the declination and inclination of geomagnetic field are −6° and 59°, respectively.

The projection of the magnetic field generated by the target in the direction of the geomagnetic field is the real signal; in addition, the geomagnetic field, UAV’s interference field, equipment noise, and power frequency interference constitute the actual signal. Gaussian white noise with different SNRs is added to the real signal as simulated synthetic signals.

### 4.2. Evaluation of Different Denoising Methods

**Figure 6:**
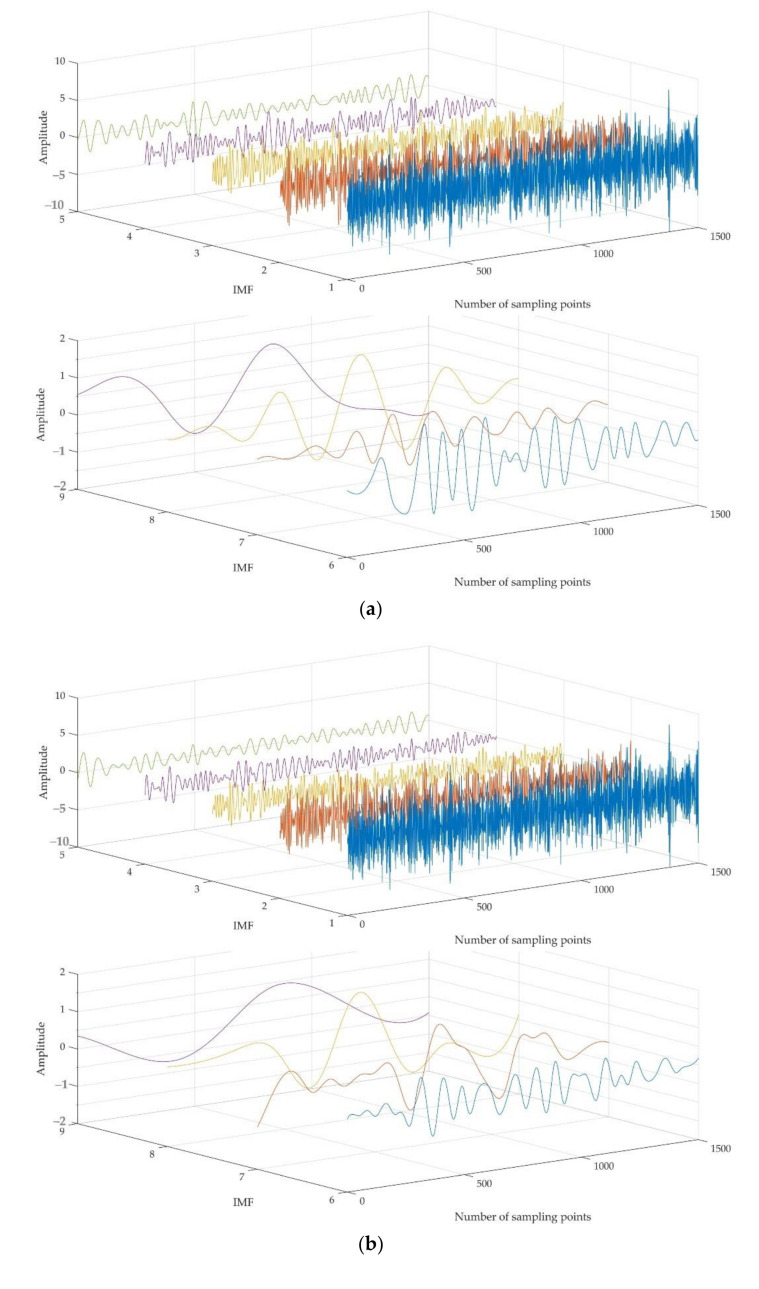

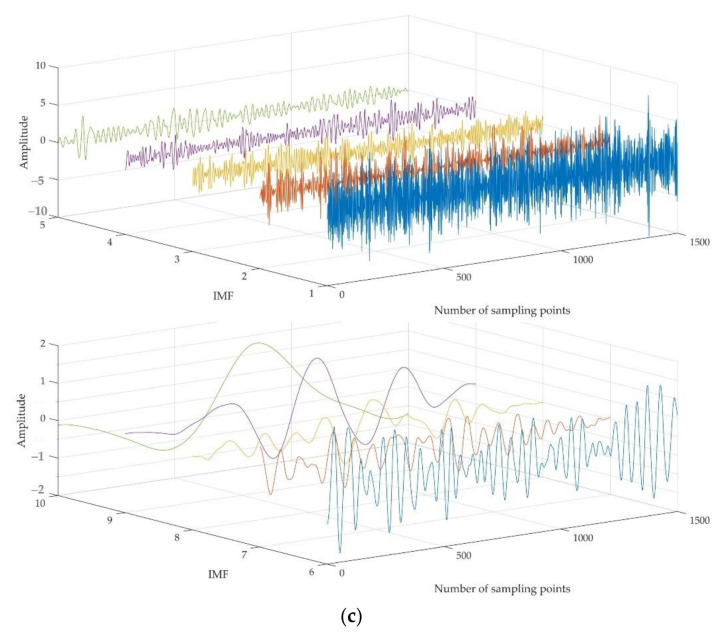
The decomposition results of the −10 dB synthetic signal by (**a**) EMD; (**b**) EEMD; and (**c**) CEEMDAN.

To clearly verify the denoising effectiveness of the proposed method, two combined noise reduction methods, i.e., EMD-PE-WSTD and EEMD-PE-WSTD, are chosen to compare with the proposed CEEMDAN-PE-CC-WSTD method. The former two methods use EMD and EEMD to decompose the original signal into several IMFs, then the noise IMFs are identified and removed using the quartile of the calculated PE. Both noise-dominant and signal-dominant IMFs are denoised using WSTD. Finally, the processed signal is obtained by combining the signal IMFs and the denoised IMFs. For EEMD and CEEMDAN-based methods, the amplitude of the added noise and the number of ensemble trials are 0.12 and 50, respectively. For the synthetic signal with a SNR of −10 dB, the decomposition results using EMD, EEMD, and CEEMDAN are shown in Figure 6. For each set of IMFs, results of PE can be obtained, as shown in Table 1.

For each decomposition method, IMFs are divided into four categories according to the corresponding quartiles of PEs, as shown in Table 2. Noise IMFs are first identified and removed. For the EMD and EEMD-based method, both noise-dominant and signal-dominant IMFs (IMF3–IMF7) are denoised using WSTD. For the CEEMDAN-based method, signal IMFs constitute the real signal, and CCs of the remaining IMFs and the real signal are obtained, as shown in Table 3. The median of the absolute value of CC sequence is 0.0232, and the IMFs corresponding to the CC greater than this value are selected as the real signal-dominant IMFs, e.g., IMF7 and IMF8 are selected in this case. Then, the real signal-dominant IMFs are denoised by WSTD, and the denoised signal can be obtained by combining the denoised IMFs and the signal IMFs.

To clearly compare different methods, SNR and root mean square error (RMSE) are used to evaluate the denoising performance. SNR shows an energy relationship between signal and noise, which is an intuitive method to evaluate the effect of the denoised signal by analyzing whether the SNR is improved. RMSE shows the difference between the denoised signal and the real target signal; the smaller the RMSE, the better the denoising effect. The formulas of SNR and RMSE are, respectively, given as follows

(24) $$SNR=10log_{10}\left(\frac{\sum_{i=1}^{n}s^{2}\left(i\right)}{\sum_{i=1}^{n}{ [s\left(i\right)-\hat{s}\left(i\right)]}^{2}}\right),$$

(25) $$RMSE=\sqrt{\frac{1}{n}\sum_{i=1}^{n}{ [s\left(i\right)-\hat{s}\left(i\right)]}^{2}},$$

where $s\left(i\right)$ is the original signal, $\hat{s}\left(i\right)$ is the denoised signal, and n is the number of sampling points.

**Figure 7:**
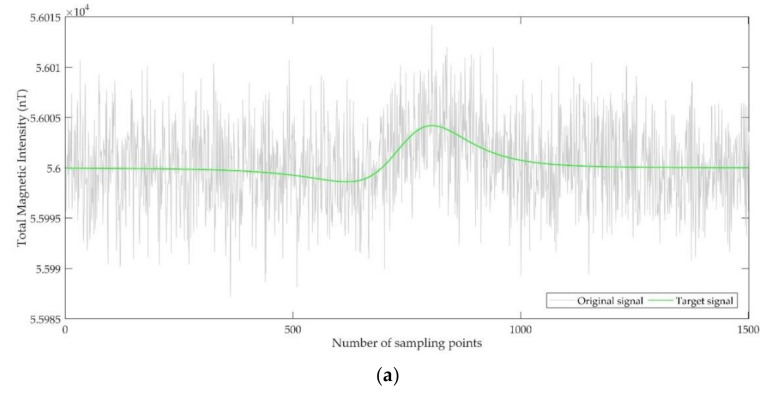

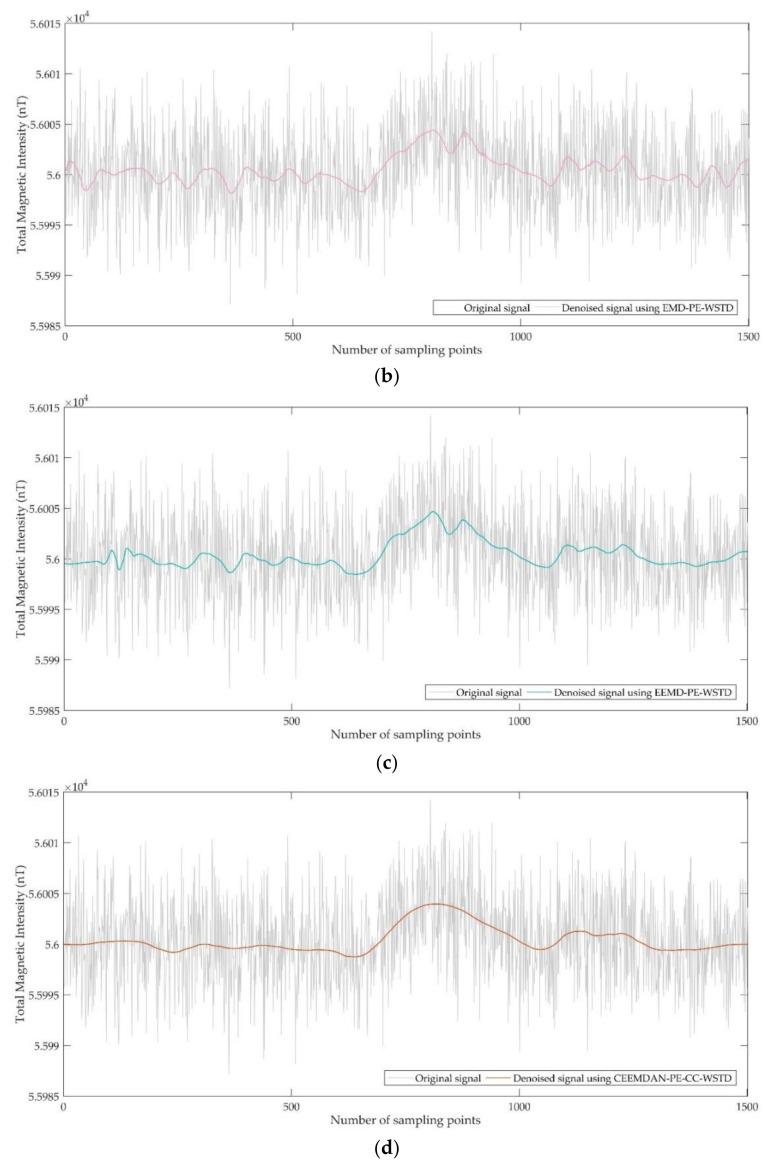
The time domain waveform before and after noise reduction of a synthetic signal with a SNR of −10 dB. (**a**) The original signal and the target signal; (**b**) the denoised signal by EMD-PE-WSTD; (**c**) the denoised signal by EEMD-PE-WSTD; and (**d**) the denoised signal by CEEMDAN-PE-CC-WSTD.

Figure 7 shows the synthetic signal with −10 dB SNR and the denoised signal based on EMD-PE-WSTD, EEMD-PE-WSTD, and CEEMDAN-PE-CC-WSTD methods. The denoised results of synthetic signals with the SNR of −10 dB, −5 dB, 0 dB, and 5 dB are shown in Table 4. As can be seen from Figure 7 and Table 4, the three denoising methods all can reduce noise, and the proposed CEEMDAN-PE-CC-WSTD method has a better performance than the other two methods. There are two main reasons to explain these results: (1) all three methods can effectively suppress noise, indicating that the quartile of PE can indeed classify IMFs into four categories according to the dominance of signal and noise, and (2) the real signal-dominant IMFs are further confirmed by the median of CC, and hence the noise in the original signal is further suppressed. It should be noted that both the classification of IMFs and the confirmation of real signal-dominant IMFs are realized through the characteristics of the signal itself, and there is no need to set a threshold artificially. The use of the quartile of PE and the median of CC makes this method completely adaptive for the analysis of noisy signals.

The WSTD alone is applied to the synthetic data with different SNRs, using two different wavelet basis functions, db4 and sym4, respectively. The decomposition level is from 3 to 7. WSTD results are shown in Table 5. It can be seen that the effect of WSTD depends heavily on the selection of wavelet basis function and the number of decomposition levels. In addition, SNR of denoised signal reaches a maximum when the decomposition level increases to a certain value. The best result of WSTD reaches the level of the EMD-PE-WSTD and EEMD-PE-WSTD method. However, it is worth noting that for the method in Table 4, we do not optimize the wavelet basis function and the number of decomposition level. Therefore, as suggested by Table 4 and Table 5, it is expected that our proposed method has a better performance than the WSTD method.

## 5. UAV Magnetic Survey Experiment Verification

### 5.1. The Multi-Rotor UAV Magnetic Survey System

**Figure 8:**
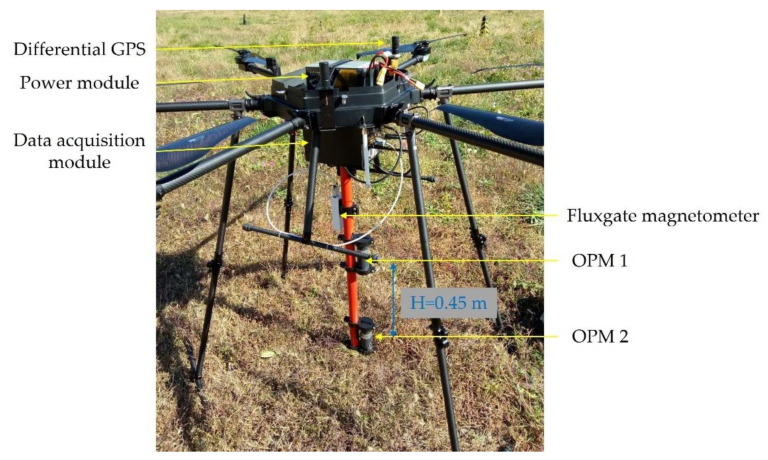
The six-rotor UAV magnetic survey system.

A multi-rotor-based UAV-magnetometer system was deployed for the purpose of near-surface targets detection, where the parameters of the system can be found in [8]. However, the magnetometers were semi-rigidly mounted below the UAV, which could not meet the requirements of vertical take-off and landing (VTOL), and the potential impact risk of magnetometers also existed [8,39]. To surmount these obstacles, a magnetic survey system based on a six-rotor UAV was developed, as shown in Figure 8. This system consisted of two cesium optically pumped magnetometers (OPMs) and a fluxgate magnetometer to record the total magnetic intensity (TMI) data and the vector magnetic intensity (VMI) data, a differential GPS to provide the location information of the UAV, a data acquisition module, and a power module. The two OPMs were rigidly mounted below the center of the UAV by a boom, with a vertical distance of 0.45 m. The TMI and VMI data were synchronized by the pulse per second signal, with a sampling frequency of 160 Hz. The technical specifications of the multi-rotor magnetic survey system are given in Table 6.

### 5.2. Evaluation of UAV-Borne Magnetic Survey Results after Denoising

**Figure 9:**
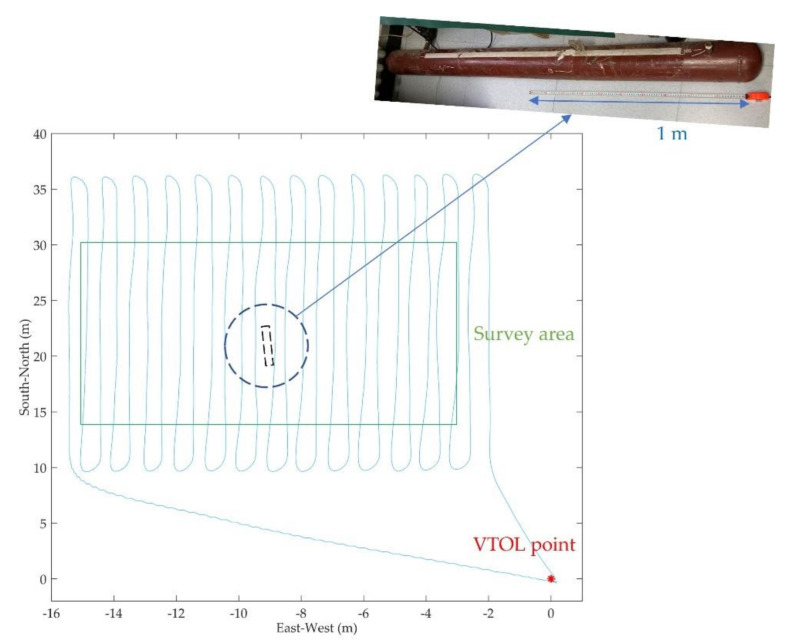
The flight profiles of the UAV and the survey area. The dotted rectangle represents the buried pipeline target.

Experiments were carried out in Sichuan, China for the detection of near-surface buried targets. A segment pipeline made of steel was used as the target with a buried depth of 3 m, a length of 2 m, and a diameter of 0.15 m. A 12 m × 16 m rectangular area was selected as the survey area, and the target was buried near the center of the survey area. The pre-programmed flight profiles ran along the north–south direction, with a line spacing of 0.5 m. Once the flight profiles were programmed, the multi-rotor UAV magnetic system was able to automatically perform survey tasks, including take-offs and landings. The flight altitude was set to 3.5 m above ground level (AGL), with a flight speed of 2 m/s. Figure 9 shows the flight profiles of the UAV. The origin of the local cartesian coordinates was the starting point of the flight. Profiles above the survey area can be obtained after cutting off the undesired and curved flight data (see Figure 9).

**Figure 10:**
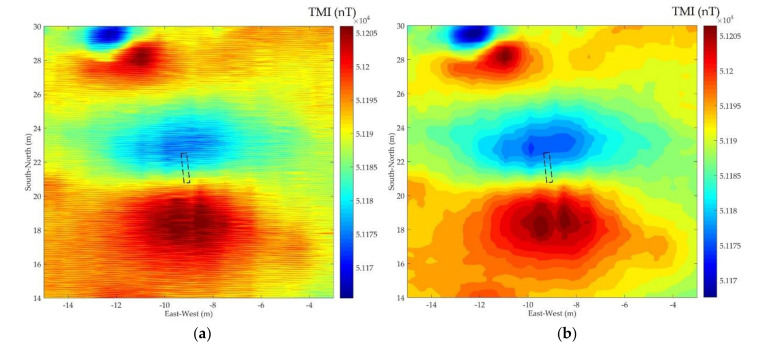

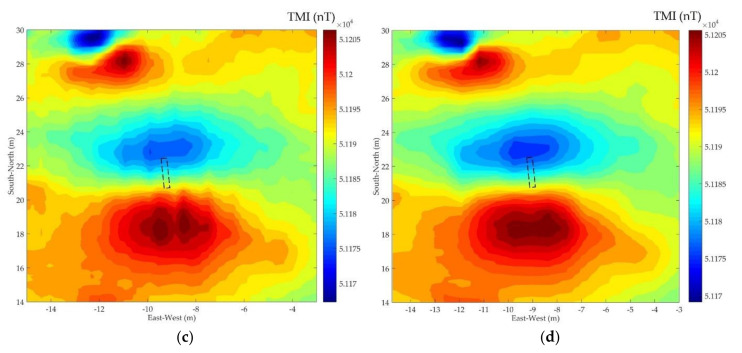
Magnetic map of the survey area by interpolating (**a**) the original data; (**b**) the denoised data by EMD-PE-WSTD; (**c**) the denoised data by EEMD-PE-WSTD; and (**d**) the denoised data by CEEMDAN-PE-CC-WSTD. The dotted rectangle represents the buried pipeline target.

The two-dimensional magnetic map of the survey area can be obtained by interpolating the original data, as shown in Figure 10a. The characteristics of the target signal were masked due to the original signal containing a lot of random noise. The denoised data of each flight profile were obtained using EMD-PE-WSTD, EEMD-PE-WSTD, and the proposed CEEMDAN-PE-CC-WSTD method, and the corresponding results of magnetic maps are shown in Figure 10b–d, respectively.

To evaluate the quality improvement of magnetic maps after denoising, we introduce two parameters, i.e., peak signal-to-noise ratio (PSNR) and structural similarity (SSIM) [40]. The target area (East–West: −12 m to −6 m, South–North: 16 m to 25 m) of Figure 10 was selected, and the reference magnetic map of the target area can be obtained using the low-frequency electromagnetic field simulation software, ANSYS Maxwell 19.0. Information about the geomagnetic field (e.g., inclination and declination) can be obtained according to the International Geomagnetic Reference Field (IGRF) model. The results of PSNR and SSIM of different denoising methods are shown in Table 7. As shown in Table 7, the results obtained by the proposed method have the largest PSNR and SSIM, therefore, the effectiveness of the proposed method is proved.

**Figure 11:**
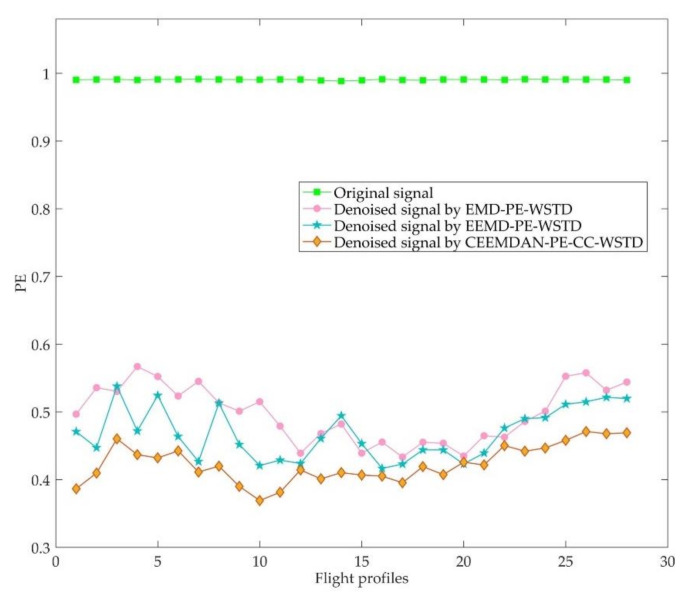
Results of PE of each flight profile data before and after noise reduction.

Considering the quasi-static characteristics of the target signal, the complexity of the flight profile data can reflect the noise level on the other hand, i.e., the flight profile data with lower PE means less noise. The results of the PE of the data of each flight profile before and after noise reduction are shown in Figure 11. The average PE of the original data, denoised data using EMD-PE-WSTD, EEMD-PE-WSTD, and CEEMDAN-PE-CC-WSTD methods are 0.9904, 0.4970, 0.4679, and 0.4231, respectively. Data denoised by the proposed method have the lowest average PE among the three methods, which indicates that the complexity of the data is significantly reduced, and the noise is greatly suppressed.

## 6. Conclusions

In this paper, a novel noise reduction method for multi-rotor UAV magnetic survey data based on CEEMDAN, PE, CC and WSTD is proposed. The CEEMDAN method is used to decompose the raw magnetic data into a series of IMFs with different scales. The quartile of PE is applied to divide the IMFs into four categories, i.e., the noise IMFs, noise-dominant IMFs, signal-dominant IMFs, and signal IMFs. Correlation coefficients are introduced to identify the real signal-dominant IMFs, and WSTD is applied to the real signal-dominant IMFs. Finally, the denoised signal can be obtained by combining the signal IMFs and the denoised IMFs. The proposed method is validated through experiments on both simulated synthetic signals and multi-rotor UAV magnetic survey data. The denoised data obtained by the proposed method are qualitatively and quantitatively analyzed and compared with EMD-PE-WSTD and EEMD-PE-WSTD methods. The results show that the proposed CEEMDAN-PE-CC-WSTD method can significantly suppress the noise and obtain a clearer target signal, which is very beneficial to the follow-up data interpretation. Our future work will include further verification of the proposed method via more UAV magnetic survey applications.

## Figures and Tables

**Table 1:** The PEs of each set of IMFs obtained by EMD, EEMD, and CEEMDAN methods.

Methods	IMF1	IMF2	IMF3	IMF4	IMF5	IMF6	IMF7	IMF8	IMF9	IMF10
EMD	0.9977	0.8768	0.7172	0.5895	0.5004	0.4510	0.4247	0.4105	0.3720	/
EEMD	0.9958	0.8792	0.7162	0.5934	0.5030	0.4556	0.4218	0.4030	0.3793	/
CEEMDAN	0.9950	0.9156	0.8249	0.7149	0.5881	0.5066	0.4781	0.4386	0.4093	0.3444

**Table 2:** Four categories of IMFs obtained by EMD, EEMD, and CEEMDAN methods.

Methods	Noise IMFs	Noise-Dominant IMFs	Signal-Dominant IMFs	Signal IMFs
EMD	IMF1, IMF2	IMF3, IMF4, IMF5	IMF6, IMF7	IMF8, IMF9
EEMD	IMF1, IMF2	IMF3, IMF4, IMF5	IMF6, IMF7	IMF8, IMF9
CEEMDAN	IMF1, IMF2, IMF3	IMF4, IMF5	IMF6, IMF7, IMF8	IMF9, IMF10

**Table 3:** Correlation coefficients between each IMF and the real signal of the CEEMDAN-based method.

Mode	IMF4	IMF5	IMF6	IMF7	IMF8
Correlation Coefficient	0.0232	−0.0114	0.0048	0.0599	0.6502

**Table 4:** The denoising results of synthetic signals using different methods.

Synthetic Signal SNR (dB)	EMD-PE-WSTD		EEMD-PE-WSTD		CEEMDAN-PE-CC-WSTD	
	SNR (dB)	RMSE	SNR (dB)	RMSE	SNR (dB)	RMSE
−15	1.8589	1.0292	2.2460	0.9763	5.0386	0.7119
−10	4.6860	0.7414	6.3507	0.6089	8.9959	0.4501
−5	9.7694	0.4209	10.1386	0.3929	11.7111	0.3348
0	15.8008	0.2060	16.0741	0.1989	18.0401	0.1596

**Table 5:** The denoising results of synthetic data using (**a**) the db4 wavelet basis function and (**b**) the sym4 wavelet basis function.

Synthetic Signal SNR (dB)	Parameter	Decomposition Level				
		3	4	5	6	7
**(a)**						
−15	SNR (dB)	−9.0441	−5.0242	−1.8074	−0.1183	−2.5242
	RMSE	3.5698	2.2473	1.5518	1.2801	1.9425
−10	SNR (dB)	−5.1317	−1.5402	0.5767	4.6652	2.5986
	RMSE	2.2885	1.5221	1.2003	0.7631	0.9763
−5	SNR (dB)	−0.1303	2.8317	5.2383	7.9447	5.9407
	RMSE	1.2872	0.9214	0.7047	0.5235	0.6713
0	SNR (dB)	6.4871	9.5743	12.3324	15.9599	14.2816
	RMSE	0.5995	0.4217	0.3090	0.2056	0.2911
**(b)**						
−15	SNR (dB)	−9.0618	−5.3393	−1.7060	−0.3396	−2.2779
	RMSE	3.5771	2.3302	1.5339	1.3131	1.6716
−10	SNR (dB)	−4.8593	−1.6571	1.1933	4.9753	2.7228
	RMSE	2.2181	1.5437	1.1197	0.7329	0.9217
−5	SNR (dB)	−0.5454	2.9740	4.9339	7.6819	5.5914
	RMSE	1.3495	0.9066	0.7306	0.5450	0.7421
0	SNR (dB)	6.5718	9.2495	12.5654	16.8108	14.4053
	RMSE	0.5937	0.4379	0.3018	0.1897	0.2830

**Table 6:** The technical specifications of the multi-rotor UAV magnetic survey system.

Module	Technical Index	Specifications
UAV	Flight speed	14 m/s, maximum air speed; 2–4 m/s, recommend speed
	Mass of payload	10 kg, maximum payload; 5 kg, standard payload
	Takeoff weight	19 kg, standard
	Endurance	35 min with a payload of 5 kg
Magnetic sensors	Cesium OPM	Operating range: 10,000 nT to 105,000 nT Noise sensitivity: 0.3 pT/sqrt (Hz) @ 1 Hz
	Fluxgate magnetometer	Operating range: ± 100 μT Noise sensitivity: 4 pT/sqrt (Hz) @ 1 Hz
Power	Data acquisition module	3200 mAh lithium polymer batterie; voltage: 24 V

**Table 7:** Results of PSNR and SSIM of different denoising methods.

Parameters	Original Data	EMD-PE-WSTD	EEMD-PE-WSTD	CEEMDAN-PE-CC-WSTD
PSNR (dB)	18.8058	29.2036	30.1307	33.6262
SSIM	0.5701	0.8600	0.8622	0.8766

## Data Availability

Data used during the study are available from the corresponding author by request.

## Abbreviations

AGL	Above ground level
CC	Correlation coefficient
CEEMDAN	Complete ensemble empirical mode decomposition with adaptive noise
EEMD	Ensemble empirical mode decomposition
EMD	Empirical mode decomposition
IGRF	International Geomagnetic Reference Field
IMF	Intrinsic mode function
OPMs	Optically pumped magnetometers
PE	Permutation entropy
PSNR	Peak signal-to-noise ratio
RMSE	Root mean square error
SNR	Signal-to-noise ratio
SSIM	Structural similarity
TMI	Total magnetic intensity
UAV	Unmanned aerial vehicle
UXO	Unexploded ordnance
VMI	Vector magnetic intensity
VTOL	Vertical take-off and landing
WSTD	Wavelet soft threshold denoising
